# Pulmonary and hemostatic toxicity of multi-walled carbon nanotubes and zinc oxide nanoparticles after pulmonary exposure in Bmal1 knockout mice

**DOI:** 10.1186/s12989-014-0061-5

**Published:** 2014-11-14

**Authors:** Katrien Luyts, Stijn Smulders, Dorota Napierska, Soetkin Van kerckhoven, Katrien Poels, Hans Scheers, Bianca Hemmeryckx, Ben Nemery, Marc F Hoylaerts, Peter H M Hoet

**Affiliations:** Department of Public Health and Primary Care, Occupational and Environmental Toxicology, KU Leuven, Leuven, Belgium; Department of Cardiovascular sciences, Center for Molecular and Vascular Biology, KU Leuven, Leuven, Belgium; Department of Public Health and Primary Care, Laboratory for Occupational and Environmental Hygiene, KU Leuven, Leuven, Belgium

**Keywords:** Bmal1^−/−^ mice, Procoagulant phenotype, Subacute study, Hemolysis, Zinc-induced inflammatory suppression

## Abstract

**Background:**

Pulmonary exposure to nanoparticles (NPs) may affect, in addition to pulmonary toxicity, the cardiovascular system such as procoagulant effects, vascular dysfunction and progression of atherosclerosis. However, only few studies have investigated hemostatic effects after pulmonary exposure.

**Methods:**

We used Bmal1 (brain and muscle ARNT-like protein-1) knockout (Bmal1^−/−^) mice which have a disturbed circadian rhythm and procoagulant phenotype, to study the pulmonary and hemostatic toxicity of multi-walled carbon nanotubes (MWCNTs) and zinc oxide (ZnO) NPs after subacute pulmonary exposure. Bmal1^−/−^ and wild-type (Bmal1^+/+^) mice were exposed via oropharyngeal aspiration, once a week, during 5 consecutive weeks, to a cumulative dose of 32 or 128 μg MWCNTs or 32 or 64 μg ZnO NPs.

**Results:**

MWCNTs caused a pronounced inflammatory response in the lung with increased cell counts in the broncho-alveolar lavage and increased secretion of interleukin-1β and cytokine-induced neutrophil chemo-attractant (KC), oxidative stress (increased ratio of oxidized versus reduced glutathione and decreased total glutathione) as well as anemic and procoagulant effects as evidenced by a decreased prothrombin time with increased fibrinogen concentrations and coagulation factor (F)VII. In contrast, the ZnO NPs seemed to suppress the inflammatory (decreased neutrophils in Bmal1^−/−^ mice) and oxidative response (increased total glutathione in Bmal1^−/−^ mice), but were also procoagulant with a significant increase of FVIII. The procoagulant effects, as well as the significant correlations between the pulmonary endpoints (inflammation and oxidative stress) and hemostasis parameters were more pronounced in Bmal1^−/−^ mice than in Bmal1^+/+^ mice.

**Conclusions:**

The Bmal1^−/−^ mouse is a sensitive animal model to study the procoagulant effects of engineered NPs. The MWCNTs and ZnO NPs showed different pulmonary toxicity but both NPs induced procoagulant effects, suggesting different mechanisms of affecting hemostasis. However, the correlation analysis suggests a causal association between the observed pulmonary and procoagulant effects.

## Background

The rapid developments in nanotechnologies have increased the production of nanoparticles (NPs). These engineered NPs are, due to their unique physico-chemical properties, currently incorporated in thousands of products in a broad range of industries. This leads to an increased risk of exposure in occupational and environmental settings [1]. Obviously, the lungs are an important target for toxicity studies since it is the main exposure route and site of particle deposition. However, the effects are probably not limited to the respiratory tract, since environmental ultrafine particles (UFP) can cause cardiovascular toxicity/morbidity and lately, similar observations have been made for NPs [2].

Epidemiological studies showed associations between environmental particulate matter exposure and atherosclerosis and an increased risk for myocardial infarction [3,4]. People living near (<245 meters) a major traffic road have an increased risk for deep vein thrombosis [5]. These effects are even more pronounced in human populations with underlying cardiovascular disease or risk factors (diabetes mellitus, hypertension, heart failure) [6-9]. Experimental animal models established that pulmonary titanium dioxide (TiO_2_) NP exposure significantly impairs endothelium-dependent vasoreactivity in rat coronary arterioles [10]. Intratracheal administration of aminated polystyrene NPs (5 mg/kg) significantly increased thrombus formation in the femoral vein in a hamster model [11]. Subchronic exposure to low doses of single-walled carbon nanotubes (SWCNT) and multi-walled carbon nanotubes (MWCNT) resulted in accelerated plaque formation in apo lipoprotein E knockout (ApoE^−/−^) mice [12,13].

Bmal1 (brain and muscle ARNT-like protein) is a basic helix-loop-helix-PAS domain-containing transcription factor and is, together with another member of this family, CLOCK, the key component of the molecular oscillator that generates circadian rhythms [14,15]. The central component of the circadian clock is located in the hypothalamic suprachiasmatic nucleus (SCN), near the optic chiasm. Environmental information (light/dark) received via the retina synchronizes the internal clock, and the SCN, in its turn, synchronizes the activity of peripheral oscillators present in every cell of an organism. These peripheral oscillators generate rhythms in gene expression, metabolism, and hormone secretion which ultimately result in rhythmic changes in physiology and behavior, presumably as an adaptation to the daily changes in the environment [16]. Mice with a dysfunctional circadian clock lacking the Bmal1 gene have been shown to have a prothrombotic state due to increased platelet aggregation and adhesion, compared to Bmal1^+/+^ mice [17]. They display vascular disease with endothelial dysfunction due to hyperactivated endothelial cells and an impaired endothelium-dependent vasorelaxant response [18,19]. Bmal1^−/−^ mice have an average lifespan of 37.0 ± 12.1 weeks and spontaneous deaths start at the age of 26 weeks. Also, some phenotypical changes associated with premature ageing are present such as the development of cataracts (starting at the age of only 18 weeks), decreased adipose and muscle tissue mass compared to Bmal1^+/+^ mice and reduced hair growth [20].

These observed phenotypes are caused by the deregulation of reactive oxygen species (ROS). Bmal1 directly regulated ROS and its deficiency leads to an excessive ROS production, leading to chronic oxidative stress [20]. The role of oxidative stress in the early onset of ageing in Bmal1^−/−^ mice has been proven by the increased life span due to the dietary supplementation of the antioxidant N-acetyl cysteine (NAC). Also the development of cataracts was attenuated, but growth retardation, reduced hair regrowth, sarcopenia and joint ossification were not affected by NAC administration suggesting the involvement or other mechanisms besides oxidative stress for the increased ageing [21]. Several clinical conditions have been linked to chronic oxidative stress, including cardiovascular disease. An excessive stimulation of NAD(P)H oxidase activity by cytokines or other agents drives the increased ROS levels and are often associated with pathological changes indicative of a deregulation of signaling cascades and/or gene expression [22].

The Bmal1^−/−^ mouse has phenotypic characteristics of people with cardiovascular disease which are driven by oxidative stress and it is therefore believed to be a good model to study extra-pulmonary toxicity such as effects on coagulation and the vasculature, after pulmonary exposure to NPs. Additional to the role of the Bmal1 gene in the cardiovascular system, the Bmal1^−/−^ mouse model previously has been used for research regarding osteogenesis, myogenesis, insulin resistance and obesity [23-25].

In order to assess whether Bmal1^−/−^ mice are more prone to the adverse effects of pulmonary administered NPs, we subacutely exposed Bmal1^−/−^ and Bmal1^+/+^ mice to MWCNT or ZnO NPs, via oropharyngeal aspiration. 24 hours or 8 weeks after the last administration, we assessed pulmonary inflammation and oxidative stress together with effects on blood cell counts, coagulation status and the early onset of vascular inflammation (macrophage influx).

## Results

### Broncho-alveolar lavage

**Figure 1:**
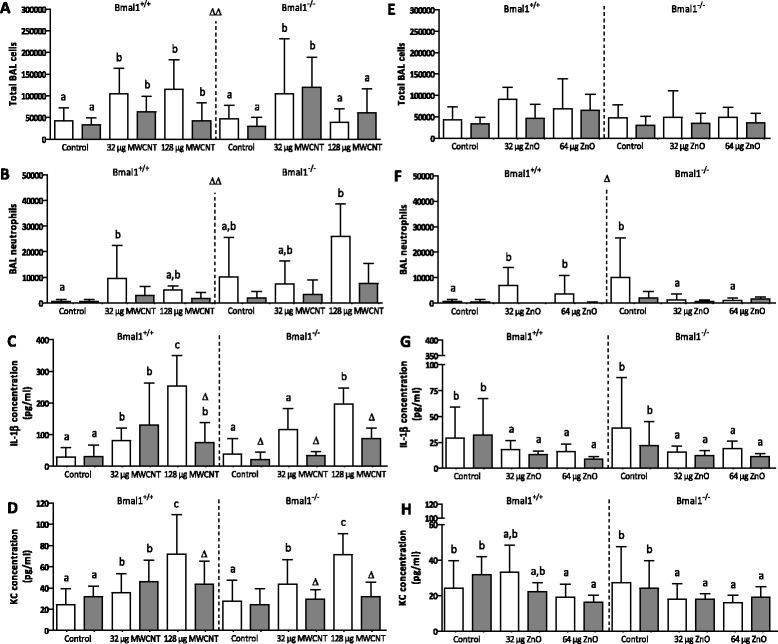
**Pulmonary inflammatory endpoints measured in Bmal1** ^**+/+**^ **and Bmal1** ^**−/−**^ **mice, 24 hours (T1) and 8 weeks (T2) after the last exposure to multi-walled carbon nanotubes or zinc oxide nanoparticles.** Total BAL cells **(A, E)**, BAL neutrophils **(B, F)**, pulmonary IL-1β concentration **(C, G)** and pulmonary KC concentration **(D, H)** were measured in each mouse. White bars depict measurements at T1, grey bars depict measurements at T2. Significant differences between the experimental groups due to the NP administration are depicted by letters (a, b and c). The effects after NP administration is indicated with letters depicted above the groups. A different letter indicates a significant effect of this group compared to the control of the same time point, within the same genotype. Time-effects are indicated by Δ. Δp < 0.05 compared to the same experimental group of T1. ΔΔp < 0.01 compared to the same experimental group of T1. When a symbol is placed in the middle of the figure, the time effects (Δ) are observed in all experimental groups.

The total amount of cells in the broncho-alveolar lavage (BAL) fluid, and the fraction of macrophages, neutrophil, eosinophils and lymphocytes herein were calculated for each mouse. In general, the total BAL cell numbers more than doubled (p = 0.001) after administration of MWCNT without difference between the two dose groups (Figure 1A). In the Bmal1^−/−^ mice, however, the total number of BAL cells was lower after the high MWCNT dose compared to the low MWCNT dose (confirmed by the concentration x genotype interaction of principal component 1; p = 0.015). The number of BAL cells, after MWCNT treatment was significantly higher after 24 hours (T1) compared to 8 weeks later (T2) (p = 0.002). The same trends were observed for the ZnO NPs, however, the differences were less pronounced and not significant (Figure 1E). The macrophages made up the largest fraction of BAL cells and followed the same pattern as the total amount of BAL cells. For the MWCNTs, the neutrophils also followed the same pattern as the total BAL cell count (Figure 1B). However, the ZnO NPs caused, at T1, a neutrophil increase in Bmal1^+/+^ mice, but a neutrophil decrease in Bmal1^−/−^ mice (concentration x genotype interaction: p = 0.04; Figure 1F). Also, the highest numbers of neutrophils were found at T1 as compared to T2 (p = 0.004 for MWCNT and p = 0.02 for ZnO NPs) and these were generally higher in the Bmal1^−/−^ mice than in Bmal1^+/+^ mice (p = 0.009 for MWCNT) (data not shown).

A principal component analysis (PCA) taking into account the cellular influx in the lung showed a positively correlation with all 5 variables (total BAL cells and the number of each cell type) and accounted for 55% and 52% of the total variability for MWCNT (p = 0.0005) and ZnO NPs (p = 0.008), respectively. Results using PCA were very similar to those of the total cell counts (data not shown).

**Figure 2:**
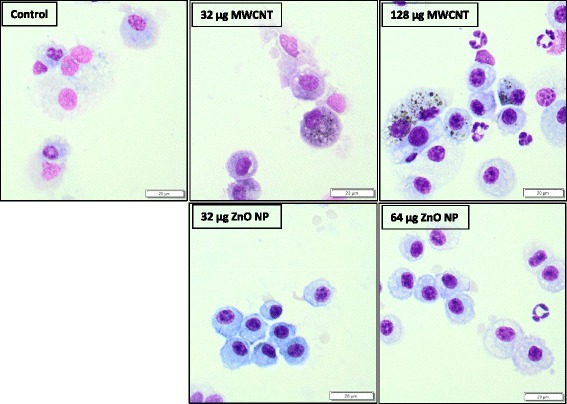
**BAL cells of Bmal1** ^**−/−**^ **mice exposed to MWCNTs and ZnO NPs.** Both groups receiving the low and high dose of MWCNT showed CNT-laden macrophages in the BAL 8 weeks after the last administration (T2) whereas the BAL cells of the mice exposed to the ZnO NPs did not show – at this magnification - any inclusions of particulate material.

The BAL macrophages show evidence of internalized MWCNTs up to T2 for both concentrations and both genotype (Figure 2). Data are only shown for the Bmal1^−/−^ mice. On the other hand, the ZnO NPs were not visible in these cells. A summary of the data can be found in Table 1.

**Table 1:** **Summary of the nanoparticle-induced, genotype and time effects**

	**MWCNT**	**ZnO**	**Genotype effect**	**Time effect**
***Pulmonary toxicity:***				
BAL cells	↑↑	≈	≈	↓ (only WT)
Lung cytokines	↑↑	↓	≈	↓
Oxidative stress	↑↑	↓	↓	↓
***Extra-pulmonary toxicity:***				
Inflammatory blood cells	≈	≈	↑	≈
Red blood cells - platelets	↓	≈	↑	↑
Coagulation	↑↑	↑	↑	≈↑
Vascular inflammation	≈	≈	≈	≈

The genotype effects represent the effects induced by both NPs in the Bmal1 −/− mice as compared to those of the Bmal1 +/+ mice; the time effects represent the effects induced by both NPs at T2 as compared to T1, without a distinction between both genotypes. ↑↑ represents a significant increase (p< 0.01) compared to its proper respectively control group; ↑ increase (p< 0.05); ↓↓ decrease (p< 0.01); ↓ decrease (p< 0.05); ≈ no significant change.

### Cytokines in lung homogenates

From the panel of pro-inflammatory cytokines measured, only the levels of IL-1β and KC were above the lower limit of detection. Administration of MWCNTs dose-dependently increased IL-1β (p < 0.001) and KC (p < 0.001) secretion in the lungs (Figures 1C and 1D). At T2 evidence of an inflammatory response was still present (increased IL-1β levels). Administration of the ZnO NPs did not have significant effects on the cytokine secretion. At T2, the KC levels in the exposed groups were decreased in the Bmal1^+/+^ mice, but increased in the Bmal1^−/−^ mice (three-way interaction, p = 0.023). A summary of the data can be found in Table 1.

### Pulmonary oxidative stress

**Figure 3:**
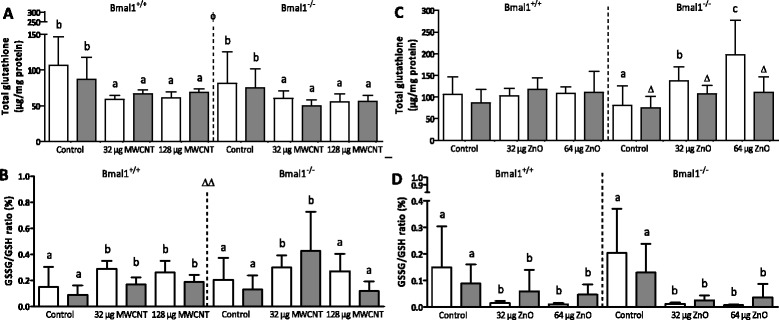
**Total glutathione and ratio of oxidized versus reduced glutathione measured in lungs of Bmal1** ^**+/+**^ **and Bmal1** ^**−/−**^ **mice, 24 hours (T1) and 8 weeks (T2) after the last exposure to MWCNTs or ZnO NPs.** Total glutathione **(A, C)** and the GSSG/GSH ratio **(B, D)** were measured in lung homogenates of each mouse. White bars depict measurements at T1, grey bars depict measurements at T2. A different letter indicates a significant effect of this group compared to the control of the same time point, within the same genotype. Δp < 0.05 compared to the same experimental group of T1. ΔΔp < 0.01 compared to the same experimental group of T1. Genotype-effects are indicated by Φ. Φp < 0.05 between Bmal1^+/+^ and Bmal1^−/−^ mice. When a symbol is placed in the middle of the figure, the time (Δ) and genotype-effects (Φ) are observed in all experimental groups.

Exposure to MWCNTs decreased the total glutathione concentrations (T1: 57.1% of the control, T2: 78.5% for Bmal1^+/+^ mice; T1: 68.2%, T2: 74.7% for Bmal1^−/−^ mice; p = 0.0001) with levels being higher for the Bmal1^+/+^ mice than the Bmal1^−/−^ mice (p = 0.034) (Figure 3A). The GSSG/GSH ratio increased after MWCNT administration (T1: 172.8%, T2: 211.4% for Bmal1^+/+^ mice, T1: 133.1%, T2: 90% for Bmal1^−/−^ mice; p = 0.005) and was higher at T1 (p = 0.003) than at T2 (Figure 3B). The ZnO NPs increased the total amount of glutathione in the lungs (T1: 101.5%, T2: 127.6% for Bmal1^+/+^ mice; T1: 242.8%, T2: 148.7% for Bmal1^−/−^ mice; p = 0.0002), with values being highest at T1 compared to T2, again only in the Bmal1^−/−^ mice (time x genotype interaction, p = 0.028) (Figure 3C). The GSSG/GSH ratio significantly decreased (Figure 3D) after ZnO NP exposure (T1: 7.1%, T2: 52.9% for Bmal1^+/+^ mice, T1: 3.3%, T2: 27.8% for Bmal1^−/−^ mice; p < 0.0001).

### Blood cell counts

The total counts of white blood cells (WBC) significantly differed between the genotypes, with the Bmal1^−/−^ mice having significantly higher WBC values compared to the Bmal1^+/+^ mice (p = 0.002 for MWCNTs and p < 0.0001 for ZnO NPs), but no effect of NP administration on the WBC levels could be detected (data not shown). The blood neutrophils, lymphocytes and eosinophils followed the same pattern (data not shown).

**Figure 4:**
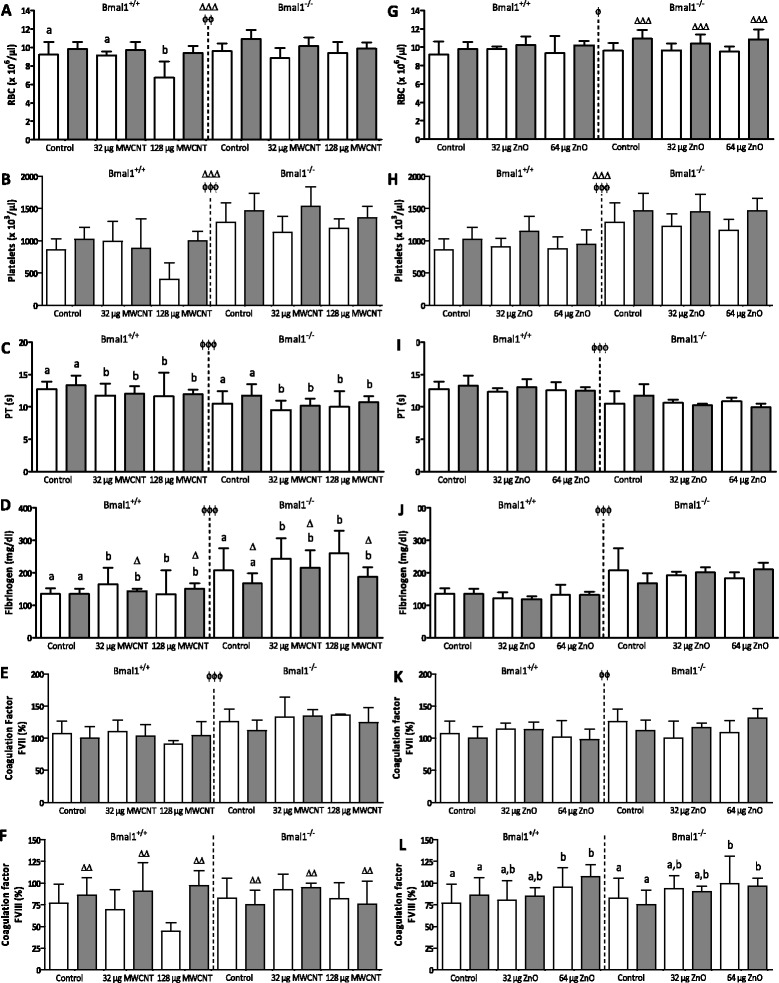
**Extra-pulmonary endpoints measured in Bmal1** ^**+/+**^ **and Bmal1** ^**−/−**^ **mice, 24 hours (T1) and 8 weeks (T2) after the last exposure to MWCNTs or ZnO NPs.** Total amount of red blood cells, (RBC) **(A, G)**, total amount of platelets **(B, H)** measured in diluted whole blood of all mice. PT **(C, I)**, fibrinogen concentrations **(D, J)**, coagulation factor VII **(E, K)** and VIII **(F, L)** measured in plasma of each mouse. White bars depict measurements at T1, grey bars depict measurements at T2. Δp < 0.05 compared to the same experimental group of T1. ΔΔp < 0.01 compared to the same experimental group of T1. ΔΔΔp < 0.001 compared to the same experimental group of T1. Φp < 0.05 between Bmal1^+/+^ and Bmal1^−/−^ mice. ΦΦp < 0.01 between Bmal1^+/+^ and Bmal1^−/−^ mice. ΦΦΦp < 0.001 between Bmal1^+/+^ and Bmal1^−/−^ mice. When a symbol is placed in the middle of the figure, the time (Δ) and genotype-effects (Φ) are observed in all experimental groups.

Overall, MWCNT decreased the number of red blood cells (RBC) in the blood (73.2% of the control), only in the Bmal1^+/+^ mice (Figure 4A). The numbers were higher at T2 than at T1 and the Bmal1^−/−^ mice had higher values than the Bmal1^+/+^ mice. Effects of dose, genotype and timepoint were not additive, as indicated by a three-way interaction effect (p = 0.035). ZnO NPs did not affect the RBC levels (Figure 4G), however the observations regarding time (p = 0.0002) and genotype (p = 0.029) were similar.

The Mean Corpuscular Volume (MCV) was however not affected by the MWCNT exposure, meaning that the hemolytic response was not accompanied by a *de novo* RBC production. The MCV was lowest in the Bmal1^−/−^ animals at T2 (time x genotype interaction: p = 0.017 for MWCNTs and p = 0.044 for ZNO NPs).

The number of platelets was not significantly affected by the administration of either NP (Figure 4B and 4H), however the mice at T2 had significantly higher platelet levels than the mice at T1 (p < 0.0001 for MWCNT and p = 0.0008 for ZnO) and the Bmal1^−/−^ mice also had more platelets (T1: 149.3% of the control, T2: 143.6%) than the Bmal1^+/+^ mice (p < 0.0001 for both NPs). The Mean Platelet Volume (MPV) correlated to the number of platelets where this parameter was higher at T1 than at T2 and also higher in the Bmal1^+/+^ mice than the Bmal1^−/−^ mice. A summary of the data can be found in Table 1.

### Hemostasis parameters

MWCNT administration decreased the PT (T1: 91.4% of the control, T2: 89.9% for Bmal1^+/+^ mice, T1: 95.3%, T2: 91.3% for Bmal1^−/−^ mice; p = 0.021), without a difference between both doses (Figure 4C). Also, the Bmal1^−/−^ mice had lower values than the Bmal1^+/+^ mice (T1: 82.5%, T2: 88.2%; for both NPs p < 0.0001). The fibrinogen concentrations were increased in the Bmal1^−/−^ mice exposed to the MWCNTs (125.5% of the control p = 0.036), but not to the ZnO NPs (Figure 4D). Overall, the Bmal1^−/−^ mice had higher fibrinogen concentrations than the Bmal1^+/+^ mice (153.1% of the control, p < 0.0001) and after MWCNT exposure the level at T1 was higher than at T2 (time x genotype interaction: p = 0.029) (Figures 4D and 4J).

Both NPs did not have an effect on the concentrations of coagulation factor FVII, however the Bmal1^−/−^ mice had higher values of this coagulation factor than the Bmal1^+/+^ mice (117.8%; p < 0.0001 for the MWCNTs and p = 0.009 for ZnO NPs). The coagulation factor FVIII was not affected by MWCNT administration. The ZnO NPs increased FVIII (T1: 123.9%, T2: 124.4% for Bmal1^+/+^ mice, T1: 120.3%, T2: 128.9% for Bmal1^−/−^ mice; p = 0.001) in both genotypes and at both time points.

In a PCA involving PT, fibrinogen, FVII and FVIII, the first PC was positively correlated with the fibrinogen concentration and coagulation factors FVII and FVIII and negatively correlated with PT, so these parameters are a good measure for coagulation. For the MWCNTs the first PC accounted for 60% of the variability and 55% for the ZnO NPs. For MWCNT administration, this first PC showed a significant dose effect (higher values for both doses than in the control group, p = 0.006), higher values at T1 than T2 (p = 0.03) and higher values in the Bmal1^−/−^ mice than in the WT (p < 0.001), which is in line with the results for PT and fibrinogen separately. For ZnO NP administration, there was only a difference for genotype (higher values in Bmal1^−/−^ mice, p = <0.001), which again confirmed the results for PT, fibrinogen and FVII. A summary of the data can be found in Table 1.

### Aorta histology

The proximal section of the aorta (aortic arch) was dissected and vertical sections were made, perpendicular to the surface of the tissue. The sections were stained for F4/80, a macrophage antigen, in order to assess early vascular inflammation. There were no significant differences in F4/80 staining between the different groups (data not shown).

### Correlations

When considering all pulmonary endpoints measured, we could observe a strong positive correlation between the BAL neutrophils and both cytokine concentrations: IL-1β (Pearson correlation coefficient (PCC): 0.696, p = 0.0007) and KC (PCC: 0.671, P = 0.0012), in the Bmal1^+/+^ mice exposed to ZnO NPs. These correlations were not present in the Bmal1^−/−^ mice, however, the IL-1β concentrations and total glutathione (PCC: 0.512, p = 0.0297) were correlated. For the MWCNT-exposed mice, there were only correlations between both cytokines in both genotypes in the Bmal1^+/+^ mice and in the Bmal1^−/−^ mice, the BAL neutrophils were correlated with both cytokines: IL-1β (PCC: 0.633, p = 0.0201) and KC (PCC: 0.665, p = 0.01232) (data not shown).

When considering all toxicological endpoints measured, we could also observe correlations between pulmonary inflammation, oxidative stress and the hemostasis parameters. In the Bmal1^+/+^ mice exposed to MWCNTs, the BAL neutrophils were positively correlated with fibrinogen concentrations in the blood (PCC: 0.644, p = 0.0095) and in the Bmal1^−/−^ mice, the IL-1β concentrations were positively correlated with fibrinogen concentrations (PCC: 0.620, p = 0.0179). In the Bmal1^+/+^ mice exposed to ZnO NPs, the total BAL cells were positively correlated with fibrinogen (PCC: 0.572, p = 0.0105).

An additional analysis was performed where all mice of the same genotype were pooled, independent of the nanomaterial they were exposed to (see Table 2). In the Bmal1^+/+^ mice, a positive correlation was found between the BAL neutrophils and fibrinogen concentrations (PCC: 0.584, p = 0.0003) and negative correlations were found between the PT and BAL neutrophils (PCC: −0.380, p = 0.0265), IL-1β concentration (PCC: −0.483, p = 0.0053) and KC concentrations (PCC: −0.426, p = 0.015). In the Bmal1^−/−^ mice, the fibrinogen concentrations were correlated with the IL-1β concentrations (PCC: 0.656, p < 0.0001), KC concentrations (PCC: 0.538, p = 0.0018) and GSSG/GSH ratio (PCC: 0.412, p = 0.019). The PT is negatively correlated with KC concentrations (PCC: −0.413, p = 0.0208).

**Table 2:** **Pearson correlation coefficiants between the pulmonary and cardiovascular endpoints measured**

**BAL total**	**BAL neutrophils**	**IL1β**	**KC**	**Total glutathione**	**GSSG/GSH ratio**	**PT**	**Fibrinogen**	
	PCC: 0.464	*PCC: 0.090*	*PCC: 0.095*	*PCC: −0.088*	*PCC: 0.120*	*PCC: −0.067*	*PCC: 0.112*	BAL total
	p = 0.0044	*p = 0.6110*	*p = 0.5914*	*p = 0.5980*	*p = 0.4739*	*p = 0.6985*	*p = 0.5167*	
	**PCC: 0.514**	**PCC: 0.408**	**PCC: 0.395**	***PCC: −0.056***	***PCC: 0.213***	***PCC: −0.172***	***PCC: 0.229***	
	**p = 0.0011**	**p = 0.0228**	**p = 0.028**	***p = 0.7607***	***p = 0.2427***	***p = 0.3153***	***p = 0.1789***	
		*PCC: 0.224*	PCC: 0.357	*PCC: −0.175*	*PCC: 0.138*	PCC: −0.380	PCC: 0.584	BAL neutrophils
		*P = 0.2103*	p = 0.0417	*p = 0.3073*	*p = 0.4222*	p = 0.0265	p = 0.0003	
		**PCC: 0.702**	**PCC: 0.675**	***PCC: −0.243***	***PCC: 0.320***	**PCC: −0.299**	**PCC: 0.319**	
		**p < 0.0001**	**p < 0.0001**	***p = 0.1800***	***p = 0.0739***	**p = 0.0765**	**p = 0.0582**	
			PCC: 0.652	PCC: −0.457	PCC: 0.541	PCC: −0.483	PCC: −0.067	IL1β
			p < 0.0001	p = 0.0066	p = 0.001	p = 0.0053	p = 0.7155	
			**PCC: 0.768**	**PCC: −0.486**	**PCC: 0.457**	***PCC: −0.229***	**PCC: −0.656**	
			**p < 0.0001**	**p = 0.0065**	**p = 0.0111**	***p = 0.2143***	**p < 0.0001**	
				*PCC: −0.121*	*PCC: 0.082*	PCC: −0.426	*PCC: −0.128*	KC
				*p = 0.4948*	*p = 0.6429*	p = 0.015	*p = 0.4845*	
				**PCC: −0.367**	**PCC: 0.319**	**PCC: −0.413**	**PCC: −0.538**	
				**p = 0.0458**	**p = 0.0857**	**p = 0.0208**	**p = 0.0018**	
					PCC: −0.715	*PCC: 0.233*	*PCC: −0.268*	Total glutathione
					p < 0.0001	*p = 0.1707*	*p = 0.1145*	
					**PCC: −0.518**	***PCC: 0.148***	***PCC: −0.311***	
					**p = 0.002**	***p = 0.4179***	***p = 0.0834***	
						*PCC: −0.121*	*PCC: 0.181*	GSSG/GSH ratio
						*p = 0.4821*	*p = 0.291*	
						***PCC: −0.163***	**PCC: 0.412**	
						***p = 0.3717***	**p = 0.019**	
							PCC: −0.446	PT
							p = 0.0064	
							**PCC: −0.727**	
							**p < 0.0001**	
								Fibrinogen

Pearson correlation coefficients (PCC) with concomitant p-values are presented for the measured endpoints. A comparison was made between the NP-exposed mice and the controls where the results of both NPs were pooled. The plain values represent the results from the Bmal1^+/+^ mice and the bold values represent the results from the Bmal1^−/−^ mice. The values in *italic* are non-significant correlations where a p-value of 0.05 was considered to be significant. No correlations were found for RBC and platelet counts, FVII and FVIII.

## Discussion

In this study we assessed the pulmonary and extra-pulmonary toxicity of MWCNTs and ZnO NPs after repeated pulmonary administrations. To our knowledge, this is the first study where Bmal1^−/−^ mice are used to study NP pulmonary toxicity and the effect on coagulation after pulmonary NP exposure.

### Nanoparticle-induced pulmonary effects

Considering the pulmonary endpoints measured, the two NPs evoked different responses; MWCNTs induced a pronounced pulmonary inflammatory response with increased BAL cells, secretion of IL-1β and KC and a clear induction of oxidative stress; in contrast, the ZnO NPs induced a different pattern of effects in the lungs, i.e. a limited cellular influx in the lung and an increased total glutathione content. The findings after MWCNTs administration are not surprising; it was shown in earlier studies that carbon nanotubes (CNTs) cause pulmonary inflammation (with granuloma formation) which can persist up to 1 month after the administration [26,27]. Here, the number of BAL cells, together with the measured cytokines, decreased over the 8 weeks after the last administration, however they were still elevated compared to the control. This is probably caused by the incomplete clearance of MWCNT which was apparent from the presence of MWCNT loaded (black) macrophages in the BAL at T2 (see Figure 2). The BAL macrophages of ZnO NP-exposed animals did not show internalized NPs, possibly because these NPs are less electron dense than MWCNTs and/or the ZnO NPs can solubilize within the time window of the experiment.

In acute inhalation studies, in general, ZnO NPs show a transient acute pulmonary inflammatory response [28,29]. A recent study also showed pulmonary inflammation after a single acute exposure (inhalation of 0.86 mg/m^3^ during 5 hours), but after inhalation of ZnO NPs at the same dose during 5 consecutive days no inflammation was found [30]. This is comparable to observations in humans after pulmonary exposure to high doses of zinc and/or zinc oxides. A syndrome known as metal fume fever (MFF) is an acute inflammatory response which resolves 1–4 days after exposure [31]. However, there are some discrepancies with regard to the dose of which MFF is triggered. Several studies reported symptoms after exposure ranging from 77 mg zinc/m^3^ (15–30 minutes) to 600 mg zinc/m^3^ (10–12 minutes), whereas concentrations slightly higher than the permissible exposure limit (5 mg/m^3^) during several hours did not elicit MFF-like symptoms [32].

The subacute exposure of ZnO NPs in this study only led to a very mild (and non-significant) total BAL cell increase 24 h after the last administration, which decreases at T2. The differences with the aforementioned studies are the method of exposure (inhalation vs. oropharyngeal aspiration) and therefore, the doses cannot easily be compared. In the current study, we chose to administer multiple times a low dose (6.4 or 12.8 μg/instillation) thus avoiding local epithelial damage in the lung and acute toxicity. This procedure might explain the lack of a significant inflammatory response, as in the study by Chen *et al*. [30]. Considering only the BAL neutrophils in the Bmal1^+/+^ mice, our data are in accordance with other reports, although a decrease in neutrophils was observed in the Bmal1^−/−^ mice, accompanied by a significant decrease of pulmonary cytokines and a significant increase in total glutathione.

Moreover, a zinc excess is known to suppress immunity. In humans, plasma-zinc occurs in a concentration of only 12–16 μmol/L but a deficiency as well as an excess cause decreased function of several immune cells [33,34]. Zinc excess causes macrophage activation, directs the chemotactic activity of neutrophils and suppression of NK cell functions. Also, T cell functions are suppressed and B cells undergo apoptosis [34]. We presume that our doses are at the border of toxicity explaining the absence of pulmonary inflammation and the suppression of cytokine release. We also cannot distinguish between effects caused by dissolved or particulate Zn. Preliminary dissolution studies showed a pronounced dissolution in water and a somewhat lower dissolution in medium [35]. The dispersion medium (sterile water) used in this study contained 2 vol% serum which can possibly reduce the dissolution (compared to water) during the preparation of the samples. Taking into account the large volume for dissolution of Zn in a vivo system it can be assumed that the ZnO NPs dosed will finally solubilize fully.

Possibly, the responsiveness of the immune system in the Bmal1^−/−^ mice differed from that of the Bmal1^+/+^ mice. Bmal1 has anti-inflammatory effects since it reduced Ccl2 transcription and attenuated Ly6Chi monocyte numbers and inflammation at an inflamed site [36,37]. Inflammatory diseases (asthma, rheumatoid arthritis, atherosclerosis) are known to have strong circadian components with exacerbations at night or during the early morning. Moreover, several experimental studies showed a worse disease outcome or higher mortality when wild-type mice were housed in constant darkness than at the normal light/dark cycles [37]. So, given these results, one would expect an increased inflammatory response in Bmal1^−/−^ mice, however, we found a similar vascular macrophage influx in both genotypes for the MWCNTs. Moreover, Hemmeryckx *et al.* showed an absence of vascular inflammatory response in the mesenteric blood vessels of Bmal1^−/−^ mice despite the high numbers of circulating leukocytes, suggesting these cells are less reactive [19].

### Nanoparticle-induced hemostasis effects

The pulmonary administration of the NPs caused hemostatic toxicity, in addition to the observed pulmonary effects. The MWCNTs showed a remarkable anemic effect with significantly decreased RBCs and non-significantly decreased platelets at the cumulative dose of 128 μg. However, these observations were absent in the Bmal1^−/−^ mice. Most research regarding hemolysis has been performed on human or animal whole blood with direct contact with the RBCs [38,39]. However, one study has shown the hemolytic effect of acid functionalized SWCNTs after intratracheal instillation in Swiss mice [40]. The effect was transient with RBC counts returning to semi-normal levels after 72 hours. As an explanation for this anemic response, the authors suggested the possibility of SWCNTs to rapidly diffuse through the thin alveolar membrane. However, we doubt that the anemic response is a direct effect since it has been shown that CNTs are cleared very poorly from the lungs and thus only a very small portion of the administered dose might eventually reach the blood stream. Elgrabli *et al*. observed the retention of 37% of the administered dose, 3 months after a single instillation of 100 μg CNTs in a rat lung [41]. Muller *et al.* observed the retention of 81.2% of the administered dose, 60 days after the instillation of 0.5 mg/rat [42].

A possible explanation for this acute anemic response is the state of “anemia of inflammation” which is an immune disorder that has been reported in numerous diseases with an inflammatory component [43]. As an example, in chronic obstructive pulmonary disease (COPD) an incidence of anemia has been reported ranging from 5 – 33%, with highest incidences during acute exacerbations [44-46]. It is believed that inflammatory cytokines interfere with normal erythropoiesis in a complex process where the dysregulation in iron homeostasis and erythropoietin production, impaired proliferation of erythroid progenitor cells and a reduced life span of RBCs are involved [47]. Here, the observed anemic effect is acute and resolves at T2, which coincides with the decreased pulmonary inflammation at T2.

The subacute administration of both NPs also caused a procoagulant effect with the MWCNTs causing a significantly shorter PT and increased fibrinogen concentrations while ZnO NPs exposure significantly increased the amount of coagulation factor VIII. Local inflammation can induce an acute phase response which is a nonspecific systemic reaction with neurological, endocrine and metabolic alterations [48]. As a consequence, several plasma proteins are induced e.g. C-reactive protein, cytokines and coagulation factors. Fibrinogen and FVIII are both acute phase proteins, associated with cardiovascular disease [49,50]. The hemostatic effects induced by MWCNTs can be explained by this reaction; however, the ZnO NPs did not cause an inflammatory response suggesting that both NPs affect hemostasis by different mechanisms. Possibly, other factors e.g. oxidized lipids and proteins can be involved activating the acute phase response and/or neurohumoral signaling, resulting in an altered cardiovascular function. It is possible that none of these mediators individually stimulate the extrapulmonary responses but together orchestrate this response [51]. Moreover, the Pearson’s correlations analysis indicated a correlation between the fibrinogen concentrations on the one hand and BAL neutrophils and IL-1β concentrations in the lungs on the other hand, for the MWCNT-exposed mice. This is suggestive for a causal relation between the pulmonary inflammation with the observed prothrombotic effects.

The hemostatic effects were more pronounced in the Bmal1^−/−^ mice, but also present in the Bmal1^+/+^ mice. Previous studies reported a prothrombotic effect after acute pulmonary administration of NPs. Jun *et al*. observed enhanced venous thrombus formation and platelet aggregation after the intratracheal instillation of 5–10 mg/kg bw silver NPs in rats [52]. Starting from a concentration of 50 μg diesel exhaust particles (DEPs), Nemmar *et al*. observed acute increased thrombosis formation in the right femoral vein and artery in hamsters [53]. On the contrary, Zhu *et al*. reported an increased PT and aPTT compared to the control, 30 days after intratracheal instillation of 0.8 mg/kg bw ferric oxide NPs in rats [54]. Yoshida *et al*. also reported an increased aPTT and marginally increased PT after the intranasal administration of 0.5 mg 30 nm and 70 nm SiO_2_ NPs during 7 days [55]. However, these doses are very high compared to the doses used in our study and are not representative for human exposure.

Nemmar *et al.* has indicated the importance of cross-talk between the pulmonary and systemic compartment in a study where the neutrophil enzyme elastase, secreted by pulmonary neutrophils, was responsible for the peripheral thrombotic effect observed in a hamster model [56]. Neutrophil elastase has been shown to promote the fibrinogen binding activity of the platelet integrin αIIbβ3 by cleaving the αIIb subunit. This cleavage potentiates platelet aggregation induced by low concentrations of cathepsin G, another neutrophil protease [57,58].

### Genotype-related effects

Along the NP-induced effects, there were also significant differences in the blood parameters between both genotypes. The Bmal1^−/−^ mice exhibit higher amounts of WBCs, RBCs and platelets, and showed a prothrombotic phenotype as evidenced by shorter PT and increased fibrinogen and coagulation FVII concentrations. These results confirm earlier reports [17,19]. Moreover, the correlations between the pulmonary endpoints (inflammation and oxidative stress) and hemostasis parameters were more pronounced in the Bmal1^−/−^ mice, than in the Bmal1^+/+^ mice, suggesting that the Bmal1^−/−^ are more sensitive to nanomaterial-induced effects, confirming our hypothesis.

A potential explanation for this prothrombotic phenotype is the deregulation of e.g. PAI-1, the most important regulator of fibrinolysis. PAI-1 is subject to diurnal variation, but in the Bmal1^−/−^ mice these levels are continuously elevated resulting in an imbalance between fibrinolysis and its inhibition [17]. On the contrary, Hemmeryckx *et al*. observed decreased PAI-1 gene expression in subcutaneous and gonadal adipose tissue [59]. Also vWF is regulated by the circadian transcription complex and Bmal1^−/−^ mice have increased vWF plasma levels, contributing to the prothrombotic phenotype in these mice.

### Time-related effects

We also observed several “time-effects”: the pulmonary parameters decreased over time whereas the RBCs, platelets and coagulation increased over time. The fact that the measured pulmonary endpoints were decreased at T2 compared to T1 is not surprising. The observed inflammatory and (anti-)oxidative responses attenuated over the 8 weeks after the last exposure which is indicative of a healing process. The changes observed regarding RBCs and platelets are quite similar compared to the findings of Hemmeryckx *et al*., however our RBC levels are higher and platelet levels are lower as compared to theirs [19]. They also reported in Bmal1^−/−^ mice a significantly decreased PT with age, significantly increased FVII and trends towards increased coagulation factor VIII and fibrinogen concentrations. Our results were less pronounced and not consistent in all experimental groups. This can probably be explained by the fact that our mice at T2 were 10 weeks younger than the mice used in the study of Hemmeryckx *et al*. and therefore the prothrombotic effects due to ageing could not yet be detected.

Lately, a lot of research focused on the progression of atherosclerosis following pulmonary NP exposure. The first step in the process of atherosclerosis is endothelial activation with an upregulation of adhesion molecules followed by the recruitment of macrophages to the vascular wall [60]. We therefore stained sections of the aorta for macrophages (F4/80 antigen) to assess whether the NPs used in this study could initiate this process of early atherogenesis. At the time points measured, we could not detect any differences in macrophage staining between the different experimental groups. However, Cao *et al.* observed increased plaque formation in ApoE^−/−^ mice (24 hours after the last instillation), using the same MWCNTs and the same dose (25.6 μg/instillation) in the same dosing scheme [12]. In the BMAL1^−/−^ model, exposure to MWCNT affected in a similar way the pulmonary inflammation and oxidative stress: an increased neutrophilic inflammation which was persistent up to 28 days after the last administration, increased IL-1β, KC and 8-isoprostane formation in the lung. The systemic effect observed in the BMAL1^−/−^ model differed significantly from that in ApoE^−/−^, indicating that presumably the Bmal1^−/−^ mice are less prone to the development of atherosclerosis compared to the dyslipidemic ApE^−/−^ mice.

## Conclusions

In a prothrombotic mouse model, Bmal1^−/−^ mice, we studied the pulmonary and hemostatic toxicity after a subacute exposure to MWCNTs and ZnO NPs. The MWCNTs induced a potent pulmonary inflammatory response, induced hemolysis and were procoagulant, shown by a reduced PT and increased fibrinogen concentrations. The ZnO NPs somewhat suppressed the pulmonary inflammatory response but significantly increased coagulation factor VIII. The procoagulant effects and correlations between the pulmonary and hemostatic endpoint were more pronounced in the Bmal1^−/−^ mice than the Bmal1^+/+^ mice. This study supports evidence that the population with cardiovascular risk factors have higher risks for the development of hemostatic toxicity after pulmonary NP exposure. However, on the basis of our data possibly also people with a disturbed circadian rhythm, such as shift workers, could have a higher risk of developing particle-related heart disease.

## Methods

### Animal model

Breeding couples of mice heterozygous for Bmal1 (100% C57BL/6 J) were kindly provided by Dr K. Esser (University of Kentucky, Lexington, KY). Female Bmal1^−/−^ mice and Bmal1^+/+^ littermates were generated in the animal facility of the KU Leuven and genotyped as described by Bunger *et al*. [61].

All animals were kept in micro-isolation cages in a temperature- and light-controlled (12-hour night/day cycle) environment and had free access to drinking water and standard chow ad libitum. All animal procedures were approved by the Ethical Committee for animal experiments of the KU Leuven and performed in accordance with the National Institutes of Health Guide for the Care and Use of Laboratory Animals (1996).

### Nanomaterials and preparation of exposure suspensions

The MWCNTs (Arkema Graphistrength C100; NM402) and ZnO NPs were (BASF Z-Cote; zinkite, uncoated, primary size: 100 nm, NM110) were obtained from the European Commission Joint Research Centre Nanomaterials Repository (http://ihcp.jrc.ec.europa.eu/our_activities/nanotechnology/nanomaterials-repository). Detailed physico-chemical characterization was performed by Kermanizadeh *et al.* [62]. The X-ray diffraction (XRD) size for the ZnO NPs ranged between 70 to >100 nm. As determined by transmission electron microscopy (TEM), the MWCNTs had a diameter of 6–20 nm and length of 700–4000 nm and the size of the ZnO NPs ranged between 20-250/50-350 nm. The Brunauer-Emmett-Teller (BET) surface area was 225 and 14 m^2^/g for MWCNTs and ZnO NPs respectively [62].

The dissolution of Zn + ions from the ZnO NP dispersions was determined within the framework of the ENPRA (engineered nanoparticle risk assessment) project. These measurements were performed at a slightly different concentration (0.32 mg/ml) than the concentrations used in this study, namely 0.25 mg/ml (6.4 μg/aspiration) and 0.51 mg/ml (12.8 μg/aspiration). At a concentration of 0.32 mg/ml, the amount of Zn + ions measured in DMEM medium (+10%FBS +1%PenStrep) and water after 24 hours were 23.76 and 54.01 ppm respectively. The estimated solubility limits were determined as 29.6 and 67.2 μg Zn/ml in medium and water respectively [35]. In our experiments, dissolution of Zn is expected to be higher than 20 μg/ml, in both dose concentration, at the time of exposure. The dissolution kinetics of ZnO in the lung has not been assessed.

The NPs were weighed and dispersed in sterile water containing 2 vol% mouse serum (dispersion medium) to create a stock concentration of 2.56 mg/ml. Mouse serum was obtained from full blood from Bmal1^+/−^ mice. Blood, taken from the inferior vena cava, was collected in Minicollect serum tubes, left at room temperature for 30 minutes and centrifuged at 2000 g for 10 minutes. The serum was collected and tested to be free of LPS (LAL assay).

The NP stock suspensions were sonicated using a Microson™ ultrasonic cell disruptor (Misonix, Newtown, USA), equipped with a 1/8” disruptor horn for 16 minutes on ice/water. Thereafter, the stock concentrations were diluted to obtain the final concentrations for exposure: 160 and 640 μg/ml MWCNT (corresponding to 6.4 and 25.6 μg/dosing) and 256 and 512 μg/ml ZnO (corresponding to 6.4 and 12.8 μg/dosing). Based on preliminary studies, we chose to lower the dose of ZnO NPs exposure since the pulmonary administration of 25 μg ZnO NPs induced an acute mortality, whereas a dose of 12.5 μg did not [63].

The NP suspensions were used within one hour after sonication.

### Study design (Experimental lay-out)

Bmal1^−/−^ and Bmal1^+/+^ mice were dosed (under light anesthesia with isoflurane) via oropharyngeal aspiration once a week, during 5 consecutive weeks. The first dose was administered at the age of 8 weeks. For the MWCNT, the Bmal1 mice were either dosed 6.4 μg per aspiration (total amount administered of 32 μg) or 25.6 μg per aspiration (total of 128 μg). For the ZnO NPs, mice were either dosed 6.4 μg or 12.8 μg per aspiration (for a total of 32 μg or 64 μg respectively). Each administration consisted of a volume of 25 μl. The control animals received the dispersion medium. Mice were sacrificed either, 24 hours after the last administration (T1, age of 12 weeks) or 8 weeks after the last administration (T2, age of 20 weeks).

The mice were sacrificed using an intraperitoneal overdose of pentobarbital. Blood was immediately taken via the retro-orbital plexus and collected in a tube containing citrate. The end concentration of citrate in the blood was 0.38%. A broncho-alveolar lavage (BAL) was performed, consisting of two flushes of 1 ml/25 g body weight. The aortic arc/bow was taken and used for histology (kept at 4% formaldehyde). Finally, the lung-heart block was taken and the superior lobe of the right lung and the heart were kept on 4% formaldehyde for histology. The left lung was snap-frozen for GSSG/GSH measurements. The remaining lobes of the right lung were also snap-frozen and kept for cytokine measurements.

### Blood samples

Immediately after the withdrawal, a small portion of the blood was diluted 5-fold (in saline) to perform cell counts using the Cell-Dyn® 3200 R counter (Abbott Diagnostics, Louvain-la-Neuve, Belgium). The remaining part of the blood was immediately centrifuged twice at 16100 g, for 10 minutes at room temperature. The plasma was stored at −80°C for measurements of the partial thromboplastin time (aPTT), the prothrombin time (PT) and plasma levels of coagulation factors (F)VII, FVIII and fibrinogen using the BCS-XP automated coagulation analyzer (Siemens, Beerzel, Belgium).

### Broncho-alveolar lavage

The pooled BAL fluid was centrifuged at 400 g, for 10 minutes at 4°C. The supernatant was stored at −80°C. The cell pellet was resuspended and a total cell count was performed using a Bürker cell counter. Cells were spun on microscope slides at 300 g for 6 minutes (Cytospin 3, Shandon, TechGen, Zellik, Belgium) and stained using the DiffQuick® method (Medical Diagnostics, Düdingen, Germany). For each sample, the number of macrophages, neutrophils, eosinophils and lymphocytes were counted.

### Cytokine release

The lung tissue was homogenized in 0.5 ml of 1% bovine serum albumin (BSA in HBSS-) and afterwards centrifuged at 16100 g, 4°C for 10 minutes. The supernatant was frozen at −80°C until cytokine measurement using the MSD mouse pro-inflammatory 7-plex (IFN-γ, IL-1β, IL-6, IL-10, IL-12p70, KC/GRO, TNF-α) according to the manufacturer’s instructions (Meso Scale Discovery, Gaithersburg, Maryland, USA).

### Glutathione determination

The left lung was homogenized on ice in cold 40 mM N-ethylmaleimide (20 ml/g tissue, to prevent rapid oxidation of GSH) and then centrifuged at 14000 g for 15 minutes at 4°C. The supernatant was transferred to a new tube and 5% metaphosphoric acid was added (1/5th of the supernatant volume, final concentration is 1% metaphosphoric acid, for removing the proteins), mixed and again centrifuged at 14000 x g during 15 minutes at 4°C. The supernatant was stored at −80°C until measurements. The measurements of the reduced (GSH) and oxidized (glutathione disulfide, GSSG) forms of glutathione were performed by ultra-pressure liquid chromatography (UPLC), in combination with tandem mass spectrometry (MS-MS). An in-house developed LC/MS-MS method was used, partially based on the method of Guan et al. as follows. The LC/MS-MS analysis was conducted on a Waters® Acquity TM UPLC coupled to a Waters® Micromass MS Technologies Quattro Premier TM mass spectrometer using electro spray ionization (ESI). The LC separation was done on a Waters Acquity UPLC BEH C18, 50 mm × 2.1 mm, 1.7 μm column, held at a temperature of 40°C. The mobile phase used for the LC separation was a mixture of 0.1% formic acid in water and 0.1% formic acid in acetonitrile. The flow rate was 0.35 ml/min and the total run time was 4 minutes. Ten μl aliquots were injected per sample. The analyses were performed in the ESI + mode and a multiple reaction monitoring (MRM) methodology was used with argon as the collision gas.

Data were normalized to protein content measured by the Bradford method using bovine serum albumin as the standard. The ratio of GSSG to total glutathione was then calculated.

### Histology

The arc/bow of the aortic tissues was embedded in paraffin, dehydrated in ethanol (70%-96%-100%) and cleared in xylene. Sections (7 μm) were vertically cut (perpendicular to the surface of the tissue) using a HM360 microtome (Microm, Walldorf, Germany). Subsequently, they were deparaffinized in xylene, followed by decreasing series of ethanol concentrations and distilled water. The sections were stained for F4/80. To expose F4/80 antigens, the sections were treated with 10 mM Na_3_-citrate with 0.05% Tween during 40 minutes at 95°C and afterwards allowed to cool. The endogenous peroxidase activity was blocked by incubating the sections in methanol supplemented with 0.3% H_2_O_2_ during 20 minutes at RT. Afterwards, the sections were incubated with TNB during 45 minutes and then the rat anti-mouse antibody (2 μg/ml, Serotec, Puchheim, Germany) was incubated overnight. The slides were incubated with the secondary antibody (rabbit anti rat Ig-biotin, DAKO) during 45 minutes and afterwards the signal was amplified using the biotinyl tyramide kit (1/50, NEL700001KT, Perkin Elmer Life Sciences, Zaventem, Belgium). F4/80 signals were visualized with 3,3'-Diaminobenzidine (DAB, Sigma-Aldrich, Diegem, Belgium).

### Statistical analysis

The associations between each biological endpoint and treatment groups were studied for MWCNT and ZnO NPs separately. Data from one single control group were used for comparison with both MWCNT and ZnO NP administration. The experimental set-up with three different variables (i.e. genotype; concentration of NP administered: control, low, high; time of measurement after administration) and all possible combinations called for a three-way ANOVA model, allowing for three-way interactions and pairwise two-way interactions between the three explanatory variables. For each endpoint, we built a full model including main effects and all two-way and three-way interaction terms. Then, non-significant interaction terms or main effects (p > 0.05) were manually removed from the model in a stepwise procedure. Whenever interaction terms involving genotype showed statistical significance, subsequent subgroup analyses were performed for Bmal1^+/+^ and Bmal1^−/−^ mice separately.

BAL cell type concentrations were highly positively intercorrelated, and so were WBC types. Therefore, in order to summarize all BAL cell or WBC types in one model (meanwhile reducing the risk of making a type I error), we performed a principal component analysis (PCA) of BAL cell types and WBC types, respectively. The first component resulting from the PCA was interpreted as an overall indicator of BAL cells or WBC augmentation and analyzed in a three-way ANOVA model similar to the one-by-one analysis of BAL cell types and WBC types.

Associations among specific biological endpoints were further explored by Pearson’s correlation analyses; initially for MWCNT and ZnO NP treatment separately and finally grouping the NP exposures per mouse phenotype.

Results are reported as the percentage of the control. Statistical analyses were performed using SAS software, version 9.3. (SAS Institute, Cary, NC, USA) and all tests were two-sided with α = 0.05.

## Abbreviations

ApoE: Apo lipoprotein E
aPTT: Activated partial thromboplastin time
BAL: Broncho-alveolar lavage
BET: Brunauer-Emmett-Teller surface area
BH4: Tetrahydrobiopterin
Bmal1: Brain and muscle ARNT-like protein – 1
BSA: Bovine serum albumin
CNT: Carbon nanotube
COPD: Chronic obstructive pulmonary disease
DAB: 3,3'-diaminobenzidine
eNOS: Endothelial nitric oxide synthase
GSH: Reduced glutathione
GSSG: Oxidized glutathione
IFN-γ: Interferon gamma
IL: Interleukin
KC: Cytokine-induced neutrophil chemo-attractant
LC: Liquid chromatography
LPS: Lipopolysaccharide
MCV: Mean corpuscular volume
MFF: Metal fume fever
MPV: Mean platelet volume
MRM: Multiple reaction monitoring
MS-MS: Tandem mass spectrometry
MWCNT: Multi-walled carbon nanotubes
NAC: N-acetyl cysteine
NO: Nitric oxide
NP: Nanoparticle
PCA: Principal component analysis
PT: Prothrombin time
RBC: Red blood cell
ROS: Reactive oxygen species
SCN: Suprachiasmatic nucleus
SWCNT: Single-walled carbon nanotubes
UPLC: Ultra-pressure liquid chromatography
T1: Time point 1 = 24 hours after the last NP administration
T2: Time point 2 = 8 weeks after the last NP administration
TEM: Transmission electron microscopy
TiO_2_: Titanium dioxide
TNF-α: Tumor necrosis factor alpha
UFP: Ultrafine particles
WBC: White blood cell
XRD: X-ray diffractogram
ZnO: Zinc oxide
